# Cigarettes smoking and e-cigarettes using among university students: a cross-section survey in Guangzhou, China, 2021

**DOI:** 10.1186/s12889-023-15350-2

**Published:** 2023-03-07

**Authors:** Hongjia Song, Xuemin Yang, Wanchun Yang, Yuxing Dai, Kun Duan, Xingtao Jiang, Guangye Huang, Min Li, Guoping Zhong, Peiqing Liu, Jianwen Chen

**Affiliations:** 1 Department of Pharmacology and Toxicology, School of Pharmaceutical Sciences, Sun Yat-Sen University, Guangzhou, 510006 Guangdong China; 2 School of Pharmaceutical Sciences, National and Local Joint Engineering Laboratory of Druggability and New Drugs Evaluation, Guangdong Engineering Laboratoty of Druggability and New Drug Evaluation, School of Pharmaceutical Sciences, Sun Yat-Sen University, Guangzhou, 510006 PR China; 3 RELX Lab, Shenzhen RELX Tech. Co, Ltd., Shenzhen, 518000 Guangdong China

**Keywords:** University students, Electronic cigarettes, Cigarettes, Smoking behavior

## Abstract

**Background:**

There is an increase in the use of cigarettes and e-cigarettes worldwide, and the similar trends may be observed in young adults. Since 2014, e-cigarettes have become the most commonly used nicotine products among young adults (Sun et al., JAMA Netw Open 4:e2118788, 2021). With the increase in e-cigarette use and the decrease in use of cigarettes and other tobacco products, however, there is limited information about Chinese smokers, e-cigarettes users and trends in cigarettes and e-cigarettes use among university students. Therefore, our objective was to investigate the using status of cigarettes, e-cigarettes and smoking behavior among the students from 7 universities in Guangzhou, China.

**Methods:**

Students at 7 different universities in Guangzhou were investigated online in 2021 through a cross-sectional survey. A total of 10,008 students were recruited and after screening, 9361 participants were adopted in our statistics. Descriptive analysis, Chi-square analysis, and multiple logistic regression analysis were used to explore the smoking status and influencing factors.

**Results:**

The average age of the 9361 university students was 22.4 years (SD = 3.6). 58.3% of participants were male. 29.8% of the participants smoked or used e-cigarettes. Among the smokers and users of e-cigarettes, 16.7% were e-cigarettes only users, 35.0% were cigarettes only users, and 48.3% were dual users.

Males were more likely to smoke or use e-cigarettes. Medical students, students from prestigious Chinese universities, and students with higher levels of education were less likely. Students with unhealthy lifestyles (e.g., drinking alcohol frequently, playing video games excessively, staying up late frequently) were more likely to smoke or use e-cigarettes. Emotion can have significant impacts on both cigarettes and e-cigarettes dual users when choosing cigarettes or e-cigarettes to use. More than half of dual users said they would choose cigarettes when they were depressed and e-cigarettes when they were happy.

**Conclusion:**

We identified factors influencing the use of cigarettes and e-cigarettes among university students in Guangzhou, China. Gender, education level background, specialization, lifestyle habits and emotion all influenced the use of cigarettes and e-cigarettes among university students in Guangzhou, China. Male, low education level, from non-prestigious Chinese universities or vocational schools, non-medical specialization, and presence of unhealthy lifestyles were influencing factors for the use of cigarettes and e-cigarettes among university students in Guangzhou and students with these factors were more likely to smoke or use e-cigarettes. Besides, emotions can influence dual users' choice of products.

This study provides more information to better understand young people's preferences for cigarettes and e-cigarettes by elucidating the characteristics of cigarettes and e-cigarettes use, as well as related influencing factors, among university students in Guangzhou. Further research involving more variables connected to the use of cigarettes and e-cigarettes will be required in our future study.

## Introduction

Despite numerous efforts to stop the tobacco epidemic, tobacco smoking is recognized as a major preventable cause of disease worldwide [1]. The 2021 Global Report on trends in the prevalence of tobacco use 2000–2025, published by the World Health Organization (WHO), states that tobacco use in any form kills and sickens millions of people every year and over 8 million people died from a tobacco-related disease in 2019 [2]. Smoking and passive smoke (exposure to second-hand smoke) are the key contributors to the mortality of specialization chronic diseases, namely, cardiovascular disease, chronic respiratory disease, and cancer [3]. The prevalence of current (at least 1 of the last 30 days) cigarettes smoking among Chinese adults reached 27.7% in 2015, making it one of the highest smoking rates in the world [4]. The health risks of smoking have attracted more and more attention, and smoking on campus has become a serious school and social problem [2, 5].

Customers are getting more worried about the physical harm as their awareness of cigarettes' dangers is increased, and they are more encouraged to choose e-cigarettes which are claimed as less harmful and can meet their needs of risk reduction [6]. Many researches have shown that e-cigarettes, although they cannot be considered safe [7], may cause less harm to the body than cigarettes [8–11]. Some cigarettes smokers are converting to e-cigarettes to avoid the effects of smoking [12].

E-cigarettes are electronic devices that deliver nicotine to the respiratory system by atomizing an aerosol of smoke containing glycerin, propylene glycol, nicotine and other additives through an electric heating element [13]. Since e-cigarettes produce much less tar, carbon monoxide, and carcinogenic ingredients such as aldehydes, acids, and phenols, the exclusive use of e-cigarettes among smokers may reduce the number of diseases caused by such ingredients [14–16]. Studies have shown that cigarettes and e-cigarettes are the most frequently used nicotine products in youth adults in the USA [17–20] and probably China. China is the world’s largest consumer of tobacco products and contributes substantially to the global burden of smoking-related diseases [21]. It is noteworthy that the use of e-cigarettes in China is far less frequent than in some European countries and the United States [22–27].

However, the health risks of e-cigarettes have not been adequately studied, data on their effects and risks on human body are limited [15, 28]. Despite the fact that using e-cigarettes is a worldwide phenomenon [29, 30], there is a paucity of data regarding the knowledge and attitude of e-cigarettes users particularly among the young adults in China [31]. Studies of cigarettes and e-cigarettes use among e-cigarettes consumers are still in their infancy, with most of them being questionnaires about basic consumer information, consumption behavior and preferences. Most survey respondents are European and American e-cigarettes consumers, and there are limited reports on Chinese e-cigarettes consumers' vaping behavior. There is an urgent need to investigate the status quo and influence factors of smoking and using e-cigarettes [32].

Therefore, we conducted a cross-sectional survey of using cigarettes and e-cigarettes to investigate the smoking behaviors among university students in Guangzhou. One of our research interests was the use of cigarettes and e-cigarettes among university students. Another focus was on the factors that influence the use of cigarettes and e-cigarettes by university students.

## Methods

### Research design and participants

A cross-sectional survey was developed in China that collected data through a self-administered online structured questionnaire from July to December 2021 among undergraduate and graduate students with different disciplinary backgrounds from 7 universities in Guangzhou. In total, 10,008 participants were recruited through WeChat, while 9361 university students completed the questionnaire with a response rate of 93.5%. The online survey was anonymous, and data were encrypted for added security protection. Before entering the online survey system, all participants reviewed and approved the electronic consent page. By prohibiting users with the same IP (Internet Protocol) address from accessing the survey more than once, duplicate entries were avoided. Incomplete surveys were not sent to the system because of a missed response reminder component that alerted participants in real time about incomplete surveys. This investigation was conducted after obtaining the approval of the Ethics Review Committee (IRB), whose approval number is SYSU202108001.

#### Sociodemographic

Participants self-reported their gender, age, race/ethnicity, levels of education, and monthly living expenses. We also distinguished the university by three types (vocational school, general universities and prestigious universities) including 7 different universities in Guangzhou, China. A separate variable was created to distinguish the specialization of participants (medical specialization or not).

#### Cigarettes and e-cigarettes use

Respondents to the survey were asked whether they had smoked or used e-cigarettes even once. Those who had ever smoked or used e-cigarettes were asked if they now smoke or use e-cigarettes. We defined current cigarettes or(and) e-cigarettes use as having smoked or(and) used e-cigarettes at least one day in the last 30 days.

Current cigarettes or(and) e-cigarettes users were asked about the age at first use of cigarettes or e-cigarettes and the product of choice for first use (cigarettes or e-cigarettes). Respondents also were asked how long they have been smoking or using e-cigarettes with the possible answers being from within a month to more than ten years. The using product of initiation (cigarettes or e-cigarettes) was asked if the respondent was a dual user.

Regarding the future choices of smokers and e-cigarettes users, the main focus was to examine whether they choose to become cigarettes only users, e-cigarettes only users or dual users in a year.

#### Lifestyles variables

Previous studies [17, 18, 33] have shown that unhealthy lifestyles such as alcohol abuse, video gaming addiction, and sleep deprivation are strongly associated with smoking or using e-cigarettes in young adults, so we added lifestyle variables to the study. Three common unhealthy lifestyles were distinguished in our questionnaire including drinking alcohol excessively, playing video games frequently and staying up late (falling asleep after 24 o’clock and getting tired next morning) frequently. We defined frequently as more than three times in a week, and excessively as play video games more than 20 h per week.

#### Related concepts and definitions

In the survey, participants were divided into four types: cigarettes only users (cigarettes smokers who currently do not use e-cigarettes), e-cigarettes only users (e-cigarettes users who currently do not use cigarettes), dual users (those who currently use both cigarettes and e-cigarettes) and non-nicotine users (those who currently do not use cigarettes and e-cigarettes).

The selected Chinese universities were classified according to their academic prominence as prestigious and non-prestigious according to the QS World University Rankings [34]. Prestigious Chinese universities refer to Sun Yat-sen University and Jinan University in this study. Non-prestigious Chinese universities include Guangzhou University of Chinese Medicine, Southern Medical University and Guangzhou City Polytechnic. Guangzhou Institute of Science and Technology and Guangzhou Huashang University are vocational schools in China.

### Statistical analysis

The categorical variables were expressed as the frequency (%), while the continuous variables were presented as mean ± SD. A single sample Kolmogorov–Smirnov test was used to test whether the data conform to normal distribution. Chi square test was used to compare categorical variables, while independent sample t-test and Mann–whitney U test were respectively used to compare the continuous variables with and without normal distribution. An analysis of multiple logistic regression was conducted to explore the relationship between using behavior of cigarettes and e-cigarettes and lifestyle. When multiple comparisons were involved, the Bonferroni method was used to correct for the test level α. All analyses were done using R software. Significant test was a bilateral test and the level of statistical significance was set at *P* < 0.05 for all the analyses.

## Results

### Participant characteristics and status of cigarettes and e-cigarettes use

Table 1 shows characteristics of participants. The final sample was composed of 9361 individuals, providing a response rate of 93.5%. In the full sample of 9361 participants, 58.3% (*n* = 5461) were male and 41.7% (*n* = 3900) were female.

**Table 1:** Sociodemographic and other characteristics of university students in Guangzhou, China

Participant characteristics	*n*	*%*
**Age (Mean ± SD)**	22.4 ± 3.6	
**Gender**		
Male	5461	58.3
Female	3900	41.7
**Ethnicity**		
Han Chinese	9010	96.3
Ethnic minorities	351	3.7
**Type of University**		
Vocational schools	1874	20.0
Non-prestigious Chinese universities	3942	42.1
Prestigious Chinese universities	3545	37.9
**School**		
Sun Yat-sen University	2702	28.9
Guangzhou University of Chinese Medicine	2075	22.2
Guangzhou City Polytechnic	1382	14.8
Guangzhou Institute of Science and Technology	923	9.9
Guangzhou Huashang University	951	10.2
Jinan University	843	9.0
Southern Medical University	485	5.2
**Education Level**		
Vocational school students	1874	20.0
Undergraduates	6242	66.7
Master students	1007	10.8
Ph.D. students	238	2.5
**Specialization**		
Philosophy	291	6.3
Economics	1478	15.8
Law	896	9.6
Education	788	8.4
Literature	725	7.7
History	204	2.2
Science	1087	11.6
Engineering	993	10.6
Agronomy	117	1.2
Medicine	1370	14.6
Management	557	6.0
Art	216	2.3
Others	339	3.6
**Cigarettes or(and) e-cigarettes use**		
Use cigarettes or e-cigarettes	2786	29.8
Not use cigarettes or e-cigarettes	6575	70.2
**Types of cigarettes or(and) e-cigarettes used**		
Cigarettes only users	975	35.0
E-cigarettes only users	464	16.7
Dual users	1347	48.3

**Fig. 1:**
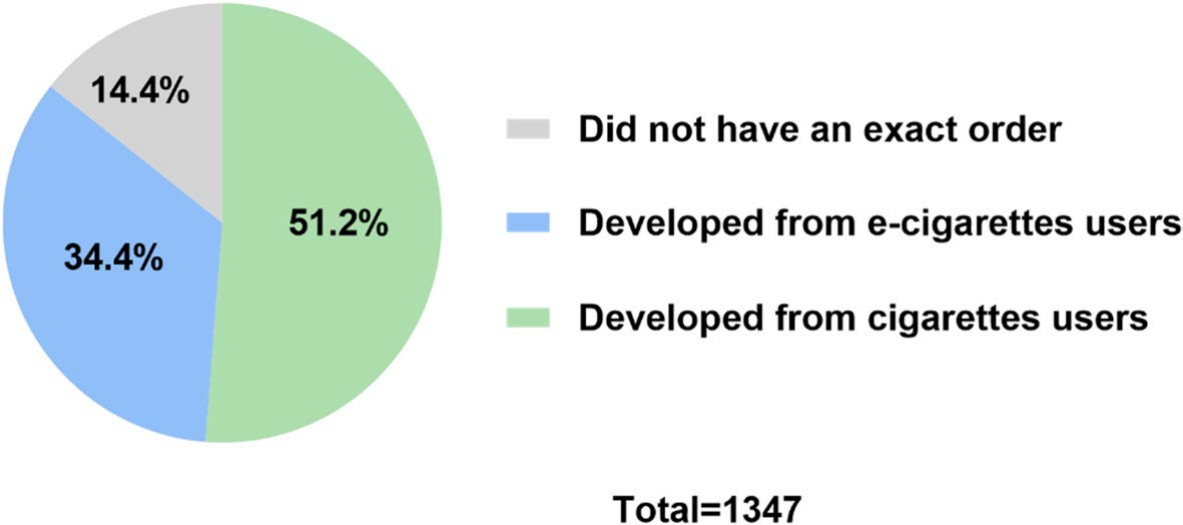
The source distribution of cigarettes and e-cigarettes dual users among university students in Guangzhou, China

Table 1 shows that 29.8% of students smoke or use e-cigarettes and that among them, the typical patterns is dual use (48.3%) with 35.0% smoking only cigarettes and 16.7% using only e-cigarettes. Among the dual users, 51.2% (*n* = 690) participants started using cigarettes, 34.4% (*n* = 464) participants e-cigarettes, 14.4% (*n* = 193) did not recall the exact order (Fig. 1).

### Factors associated with smoking or using e-cigarettes

Table 2 shows factors associated with smoking or using e-cigarettes. Among e-cigarettes users, females were more likely to choose e-cigarettes compared to males (78.1 vs. 62.8%, *P* < 0.05).

**Table 2:** Possible influencing factor related to the use of cigarettes or(and) e-cigarettes among university students in Guangzhou, China

Variable	Cigarettes or(and) e-cigarettes users			Non-nicotine products users	*χ2*	*p*-value
	Cigarettes only users	E-cigarettes only users	Dual users			
**Gender**					1251.6	< 0.05
Male	888 (16.3)	359 (6.6)	1142 (20.9)	3072 (56.2)		
Female	87 (2.2)	105 (2.7)	205 (5.3)	3503 (89.8)		
**School type**					232.6	< 0.05
Prestigious Chinese universities	312 (8.8)	104 (2.9)	339 (9.6)	2790 (78.7)		
Non-prestigious Chinese universities	445 (11.3)	261 (6.6)	730 (18.5)	2506 (63.6)		
Vocational schools	218 (11.6)	99 (5.3)	278 (14.8)	1279 (68.2)		
**Education level**					243.5	< 0.05
Vocational school students	218 (1 1.6)	99 (5.3)	278 (14.8)	1279 (68.2)		
Undergraduates	690 (11.1)	353 (5.7)	1002 (16.1)	4197 (67.2)		
Master students	42 (4.2)	9 (0.9)	47 (4.7)	909 (90.3)		
Ph.D. students	25 (10.5)	3 (1.3)	20 (8.4)	190 (79.8)		
**Specialization**					221.0	< 0.05
Non-medical	900 (11.3)	440 (5.5)	1270 (15.9)	5381 (67.3)		
Medical	75 (5.5)	24 (1.7)	77 (5.6)	1194 (87.2)		
**Lifestyles**					1151.9	< 0.05
Drinking	166 (17.0)	79 (17.0)	198 (14.7)	193 (2.9)		
Playing games	233 (23.9)	159 (34.3)	381 (28.3)	1239 (18.8)		
Staying up late	219 (22.5)	107 (23.1)	342 (25.4)	2681 (40.8)		
All of the above	277 (28.4)	78 (16.8)	361 (26.8)	998 (15.2)		
None of the above	80 (8.2)	41 (8.8)	65 (4.8)	1464 (22.3)		
**Medical specialization or not**					218.7	< 0.05
Non-medical	5381 (67.3)			2610 (32.7)		
Medical	1194 (87.2)			176 (12.8)		
**School type**					208.8	< 0.05
Prestigious Chinese universities	755(21.3)			2790(78.7)		
Non-prestigious Chinese universities	1436(36.4)			2506(63.6)		
Vocational schools	595(31.8)			1279(68.2)		
**Major**					692.9	< 0.05
Medicine	176(12.8)			1194 (87.2)		
Management	94(16.9)			463 (83.1)		
Art	45(20.8)			171 (79.2)		
Engineering	216(21.8)			777 (78.2)		
Science	239(22.0)			848 (78.0)		
Literature	201(27.7)			524 (72.3)		
Education	318(40.4)			470 (59.6)		
Economics	613(41.5)			865 (58.5)		
Agronomy	51(43.6)			66 (56.4)		
Philosophy	259(43.8)			332 (56.2)		
History	94(46.1)			110 (53.9)		
Law	423(47.2)			473 (52.8)		
Others	57(16.8)			282 (83.2)		

In general, medical students have a higher level of knowledges about health [16, 35] and it is important to understand their perceptions of e-cigarettes as they need to communicate and interact with patients during their training and later in their careers. Therefore, we divided the specialization into non-medical specialization and medical specialization, using medicine as a criterion.

The prevalence of cigarettes and e-cigarettes was significantly higher among non-medical specialization than medical specialization (32.7% vs. 12.8%, *P* < 0.05), and the highest rate of cigarettes and e-cigarettes use was found among law specialization compared to medical specialization (47.2% vs. 12.8%, *P* < 0.05), followed by history (46.1% vs. 12.8%) and philosophy (43.8% vs. 12.8%, *P* < 0.05).However, there was no difference in the choice of cigarettes or e-cigarettes between non-medical and medical students.

The use of both e-cigarettes and cigarettes was lower in prestigious Chinese universities compared to other types of schools. Students in non-prestigious Chinese universities had the highest rate of cigarettes and e-cigarettes use and a correspondingly higher rate of e-cigarettes use.

Among the participants, undergraduates and vocational school students had the highest rate of cigarettes and e-cigarettes use (32.8% and 31.8%), followed by Ph.D. students (20.2%), while master students had the lowest rate of cigarettes and e-cigarettes use at 9.7%, with a statistically significant difference (*P* < 0.05).

Among them, there was no difference in the distribution of cigarettes and e-cigarettes use among undergraduates and vocational school students, while the rate of cigarettes use among master students was significantly lower than other students (*P* < 0.05), and the rate of e-cigarettes use? was also the lowest.

Lifestyles have significant impacts on the use of cigarettes and e-cigarettes. Compared to those with appropriate lifestyles, students who drank alcohol frequently, played video games excessively, stayed up late frequently, and did all of the above had an increased odds of cigarettes use, e-cigarettes use, and dual use. Multiple logistic regression analyses of cigarettes only users, e-cigarettes only users, and dual users indicated that the using of cigarettes, e-cigarettes and dual use increased 8.1, 6.8 and 10.2 times respectively for those who drank alcohol compared to those who did not drink alcohol; The odds of cigarettes using, e-cigarettes using and dual using were 2.6, 3.2, and 4.7 times higher for gamers compared to non-gamers, respectively; The odds of cigarettes using, e-cigarettes using and dual using increased by 1.3, 1.2 and 2.4 times respectively for those who stayed up late compared to those who did not stay up late. All results are presented in Table 3.

**Table 3:** Multifactorial logistic regression assessing the effect of lifestyles on using cigarettes or(and) e-cigarettes

Variable	*OR [95% CI]*		
	Cigarettes only users	E-cigarettes only users	Dual users
None of the following	1.0	1.0	1.0
Drinking	8.1 [5.9,11.2]^*^	6.8 [4.4,10.3]^*^	10.2 [7.3,14.2]^*^
Playing games	2.6 [2.0,3.4]^*^	3.2 [2.2,4.6]^*^	4.7 [3.6,6.3]^*^
Staying up late	1.3 [1.0,1.7]^*^	1.2 [0.8,1.8]	2.4 [1.8,3.2]^*^
All of the above	3.7 [2.8,4.9]^*^	2.0 [1.3,2.9]^*^	5.6 [4.2,7.4]^*^

^*^P < 0.05

Table 4 shows that 83.5% (*n* = 1125) of dual users chose using products (whether cigarettes or e-cigarettes) according to their emotional state, while 56.5% (*n* = 761) of dual users chose cigarettes when they are depressed and e-cigarettes when they are happy.

**Table 4:** Effects of emotion on products adjustment of cigarettes and e-cigarettes dual users among university students in Guangzhou, China

Whether to choose nicotine products according to emotion	*n*	*%*
Yes, use cigarettes when you're down and e-cigarettes when you're happy	761	56.5
Yes, use e-cigarettes when you're down and cigarettes when you're happy	364	27
No, do not choose nicotine products according to emotion	222	16.5

### Future choices of smokers and e-cigarettes users

**Fig. 2:**
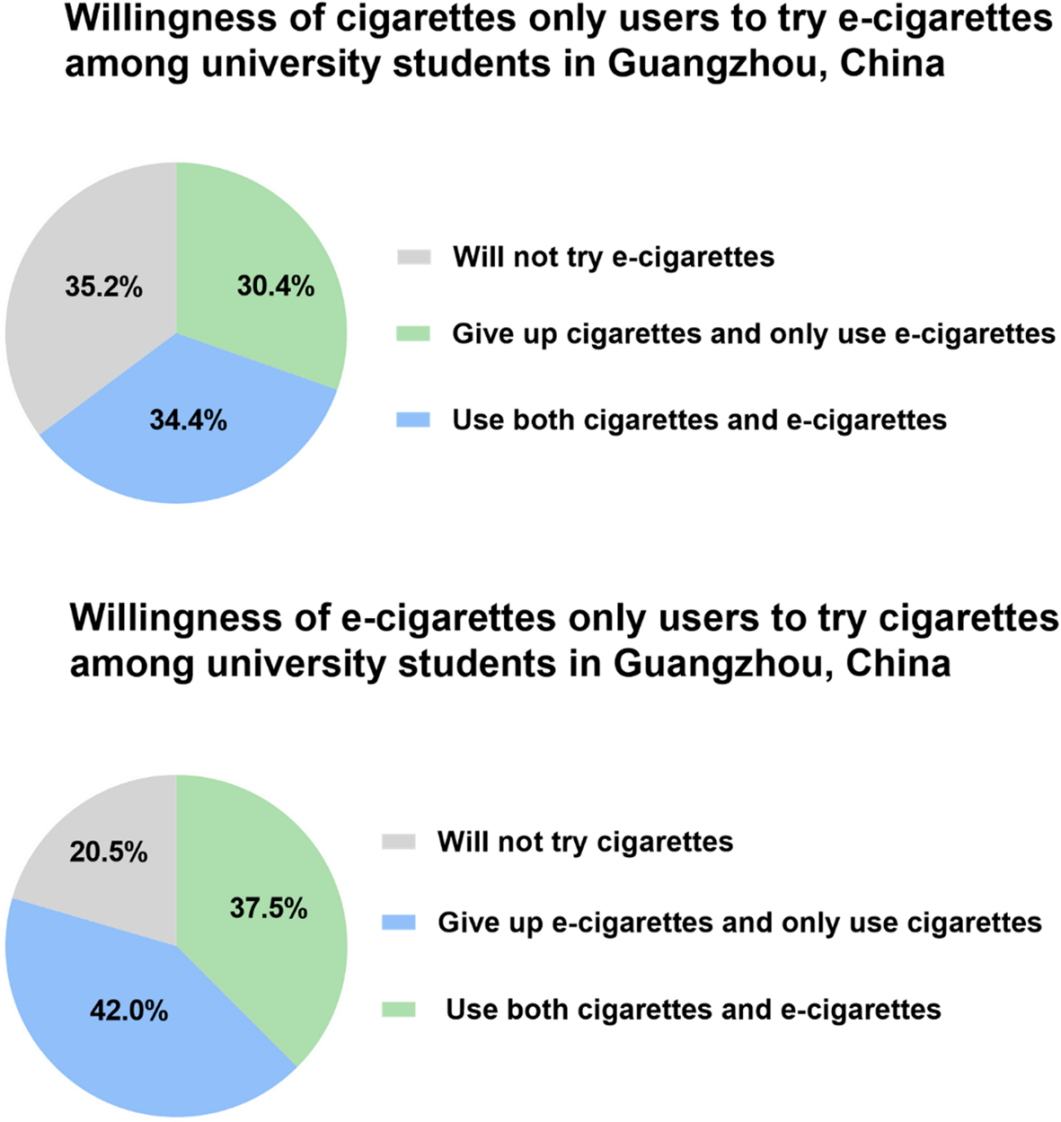
Willingness of cigarettes only users or e-cigarettes only users to try another nicotine product among university students in Guangzhou, China

Table 5 shows that e-cigarettes only users and dual users have a stronger intention to quit using their current nicotine product of use than cigarettes only users(*P* < 0.05). Figure 2 displays the willingness of cigarettes only users or e-cigarettes only users to try another product (cigarettes or e-cigarettes) among university students in Guangzhou, China. For cigarettes only users, 41.8% (*n* = 408) report that they will not use e-cigarettes in the future, 30.9% (*n* = 301) use both cigarettes and e-cigarettes in the future, and 27.3% (*n* = 266) would give up cigarettes and use e-cigarettes. For e-cigarettes only users, 42.0% (*n* = 195) would give up e-cigarettes and only use cigarettes, 37.5% (*n* = 174) would use both cigarettes and e-cigarettes, and 20.5% (*n* = 95) would not use cigarettes in the future.

**Table 5:** Percentage of intention to quit using among cigarettes or(and) e-cigarettes users among university students in Guangzhou, China

The types of cigarettes or(and) e-cigarettes users	Have willingness to quit using	Don't have willingness to quit using	*χ2*	*p*-value
			37.1	< 0.05
Cigarettes only users	770 (79.0)	205 (21.0)		
E-cigarettes only users	412(88.8)	52(11.2)		
Dual users	1175 (87.2)	172 (12.8)

## Discussion

Our findings were consistent with some prior prevalence studies in which males were more likely to smoke than females (males: females = 37.2:7.5) [10, 36–38]. However, the gender difference in e-cigarettes were smaller than in cigarettes, which is also consistent with previous research studies [39–41].

The gender differences in smoking may be attributed to traditional sociocultural influences [31, 32, 42–44]. Habitual thinking suggests that female's smoking is associated with an inappropriate social image. The social circumstances put more pressure on female smokers, whereas, for male smokers, social opinion has a much smaller negative impact than for females, suggesting that the socio-cultural context have an intervening role in smoking.

In addition, we found that the rate of using cigarettes and e-cigarettes was the highest among undergraduates, followed by Ph.D. students, and the lowest was among master students, both for cigarettes, e-cigarettes, and dual use. It indicates that cigarettes and e-cigarettes use was shown as a non-linear relationship with education level, which is consistent with other studies [45]. This may be due to the fact that undergraduates have less academic stress and more social activities [46], which are susceptibility factors for cigarettes and e-cigarettes use. A number of studies have shown that there is a significant correlation between smoking and the education level of the smoker, the higher the education level is, the lower the smoking rate is [47, 48]. This is because people with a higher level of education level have a higher level of health awareness, and a relatively higher level of awareness of the diseases caused by smoking and harmful results [49, 50], and thus have a lower smoking rate, which explains the relatively lower rate of cigarettes and e-cigarettes use among master student s and Ph.D. students. Undergraduate students were more likely to use e-cigarettes, in contrast to master students and Ph.D. students, who had the lowest rates of cigarettes and e-cigarettes use and a greater preference for cigarettes. It has been established that e-cigarettes use shows a non-linear relationship with education level, but the exact reasons for this are unclear and warrant further study [51]. Our findings displayed that the use of both e-cigarettes and cigarettes was lower in prestigious Chinese universities perhaps due to the widely different circumstances, different management, and different type of student in different universities. In addition, we found that the cigarettes use rate of Ph.D. students is much higher than the e-cigarettes use rate, which is different from the situation of undergraduates and vocational school students. The reasons for this may be that Ph.D. students are older than others and e-cigarettes are emerging products, so many Ph.D. students are used to using cigarettes and are not familiar or are not willing to try e-cigarettes.

Similar to previous surveys, we found that non-medical students have higher rates of cigarettes and e-cigarettes use than medical students [52]. This may be due to the fact that medical students are more aware of the effects of nicotine on the body after learning extensive knowledge of physiology and pathology [16, 30, 43]. It is noteworthy that, the highest rate of using cigarettes and e-cigarettes was law students. The considerable pressure placed on them in academic performance can explain this result [53].

A growing body of research indicates that emotion is also one of the influencing factors of smoking and negative emotions can induce smoking [54, 55]. Our findings found that the majority of dual users will use cigarettes rather than e-cigarettes when they are depressed. This result may be due to the different experiences of smoking and vaping while there is no related data to illustrate that smoking cigarettes will provide more pleasure in the present.

We also discovered that among all the future choices, dual use is becoming increasingly popular, as the previous study reported [56]. 51.2% of the dual users started as cigarettes only users, indicating a huge shift of nicotine products using pattern in young adults. Consistent with our findings above, some studies [30, 41, 57] also indicated that cigarettes only users are more likely to try e-cigarettes than non-smokers. However, a study by Sean Esteban McCabe et al. indicated that dual users had the greatest risk for engaging in risk behaviors (including truancy, grade point average <  = C + , binge drinking, alcohol use, marijuana use, illicit drug use and nonmedical Rx drug use) followed by cigarettes only users, e-cigarettes only users, and non-nicotine products users [5].

### Limitations

There are several limitations to this study. First, the source of the sample was university students, whose smoking behaviors may differ from the general population of young adults and may not apply to the group who are not students. Second, the data was not weighted for adjusting biases to non-equal probability of selection, non-coverage, and non-response. Third, these data are self-reported and might be subject to reporting bias. Finally, the study was a cross-sectional study and could not dynamically observe changes in cigarette and e-cigarette use, we were unable to assess causal relationships.

## Conclusion

The present study reveals the use rate of cigarettes and e-cigarettes among university students in Guangzhou, China. This study also provides the possible future choices of cigarettes or e-cigarettes users among university students. Our investigation shows that 29.8% of participants reports that they used cigarettes or e-cigarettes. Among them, 16.7% were e-cigarettes only users, 35.0% were cigarettes only users and 48.3%were dual users. 51.2% of the dual users were developed from cigarette only users.

Additionally, this study investigated influencing factors to cigarettes and e-cigarettes use, showing that gender, school, education level, specialization, and lifestyles all had impacts on the use of cigarettes and e-cigarettes among university students in Guangzhou. Students who were male, had low education levels, from non-prestigious Chinese universities or vocational schools, had non-medical specialization, and the presence of inappropriate lifestyles such as drinking and playing video games excessively were more likely to use cigarettes and e-cigarettes. Besides, emotion also can have significant effects on the choice of using cigarettes or e-cigarettes for dual users.

This study elucidates the characteristics of cigarettes and e-cigarettes use and related influencing factors among university students in Guangzhou, providing more information to better understand young people's preferences for cigarettes and e-cigarettes. This cross-section survey offers a perspective for policy makers to develop more guiding industry rules of young adult's cigarettes and e-cigarettes using.

In our future work, further investigations, which take more variables related to cigarettes and e-cigarettes using into account, will need to be undertaken, and more reliable analytical methods must be required.

## Data Availability

All data generated or analyzed during this study are included in this published article.
